# Invasive Hemodynamics and Risk Stratification in T-TEER: Moving Beyond ESC Thresholds - EuroTR Registry Insights

**DOI:** 10.1161/CIRCINTERVENTIONS.125.015964

**Published:** 2025-11-14

**Authors:** Giulia Masiero, Federico Arturi, Sara Ceni, Andrea Panza, Karl-Patrik Kresoja, Jennifer von Stein, Vera Fortmeier, Benedikt Koell, Wolfgang Rottbauer, Mohammad Kassar, Bjoern Goebel, Paolo Denti, Paul Achouh, Tienush Rassaf, Manuel Barreiro-Perez, Peter Boekstegers, Andreas Rück, Monika Zdanyte, Marianna Adamo, Flavien Vincent, Philipp Schlegel, Sebastian Rosch, Mirjam G. Wild, Christian Besler, Stefan Toggweiler, Stephanie Brunner, Julia Grapsa, Tiffany Patterson, Holger Thiele, Tobias Kister, Alessandro Sticchi, Marco De Carlo, Fabian Voss, Amin Polzin, Antonio Popolo Rubbio, Francesco Bedogni, Thorald Stolte, Thomas Nestelberger, Tomás Benito-González, Enrique Sánchez-Muñóz, Mathias H. Konstandin, Eric Van Belle, Marco Metra, Tobias Geisler, Rodrigo Estévez-Loureiro, Amir Abbas Mahabadi, Nicole Karam, Francesco Maisano, Philipp Lauten, Fabien Praz, Mirjam Kessler, Daniel Kalbacher, Volker Rudolph, Christos Iliadis, Philipp Lurz, Jörg Hausleiter, Lukas Stolz, Giuseppe Tarantini, on behalf of the EuroTR Investigators, Karl-Philip Rommel

**Affiliations:** Department of Cardiology, Cardiology I, University Medical Center of the Johannes Gutenberg-University Mainz, Mainz, Germany; Department of Cardiology, Cardiology I, University Medical Center of the Johannes Gutenberg-University Mainz, Mainz, Germany; Department of Cardiology, Heart Center, University of Cologne, Cologne, Germany; Department of Cardiology, Heart Center, University of Cologne, Cologne, Germany; Department of Cardiology, Heart Center, University of Cologne, Cologne, Germany; (Clinic for General and Interventional Cardiology/Angiology, Herz- und Diabeteszentrum NRW, Universitätsklinik der Ruhr-Universität Bochum, Med. Fakultät OWL (Universität Bielefeld), Bad Oeynhausen, Germany; Clinic for General and Interventional Cardiology/Angiology, Herz- und Diabeteszentrum NRW, Universitätsklinik der Ruhr-Universität Bochum, Med. Fakultät OWL (Universität Bielefeld), Bad Oeynhausen, Germany; Clinic for General and Interventional Cardiology/Angiology, Herz- und Diabeteszentrum NRW, Universitätsklinik der Ruhr-Universität Bochum, Med. Fakultät OWL (Universität Bielefeld), Bad Oeynhausen, Germany; Department of Cardiology, University Heart and Vascular Centre Hamburg, Hamburg, Germany; German Center of Cardiovascular Research (DZHK), Partner Site Hamburg/Kiel/Lübeck, Germany; Department of Cardiology, University Heart and Vascular Centre Hamburg, Hamburg, Germany; German Center of Cardiovascular Research (DZHK), Partner Site Hamburg/Kiel/Lübeck, Germany; Department of Cardiology, University Heart Center Ulm, Ulm, Germany; Department of Cardiology, University Heart Center Ulm, Ulm, Germany; Department of Cardiology, University Heart Center Ulm, Ulm, Germany; Department of Cardiology, Heart Center, Zentralklinik Bad Berka, Bad Berka, Germany; Department of Cardiology, Heart Center, Zentralklinik Bad Berka, Bad Berka, Germany; Cardiology Department, Centre Hospitalier Universitaire De Lille, Lille, France; Cardiology Department, Centre Hospitalier Universitaire De Lille, Lille, France; University Hospital Essen, University of Duisburg-Essen, West German Heart and Vascular Center, Department of Cardiology and Vascular Medicine, Essen, Germany; Hospital Álvaro Cunqueiro, Vigo, Spain; Hospital Álvaro Cunqueiro, Vigo, Spain; Medical Clinic III, University Hospital Tübingen, Tübingen, Germany; ASST Spedali Civili di Brescia and Department of Medical and Surgical Specialties, Radiological Sciences, and Public Health, University of Brescia, Brescia, Italy; ASST Spedali Civili di Brescia and Department of Medical and Surgical Specialties, Radiological Sciences, and Public Health, University of Brescia, Brescia, Italy; ASST Spedali Civili di Brescia and Department of Medical and Surgical Specialties, Radiological Sciences, and Public Health, University of Brescia, Brescia, Italy; ASST Spedali Civili di Brescia and Department of Medical and Surgical Specialties, Radiological Sciences, and Public Health, University of Brescia, Brescia, Italy; Cardiology Department, Centre Hospitalier Universitaire De Lille, Lille, France; Cardiology Department, Centre Hospitalier Universitaire De Lille, Lille, France; Cardiology Department, Centre Hospitalier Universitaire De Lille, Lille, France; Department of Internal Medicine III, Division of Cardiology, University Hospital Heidelberg, Ruprecht-Karl University Heidelberg, Heidelberg, Germany; Department of Internal Medicine III, Division of Cardiology, University Hospital Heidelberg, Ruprecht-Karl University Heidelberg, Heidelberg, Germany; University Heart Center Freiburg/Bad Krozingen, Bad Krozingen, Germany; Cardiothoracic and Vascular Department, Azienda Ospedaliero-Universitaria Pisana, Pisa, Italy; Cardiothoracic and Vascular Department, Azienda Ospedaliero-Universitaria Pisana, Pisa, Italy; Department of Cardiac Surgery, University Hospital Basel, University of Basel, Basel, Switzerland; Department of cardiac, thoracic vascular sciences and public health, University of Padua, Padua, Italy; Medizinische Klinik und Poliklinik I, LMU Klinikum, LMU München, Munich, Germany; Medizinische Klinik und Poliklinik I, LMU Klinikum, LMU München, Munich, Germany; Medizinische Klinik und Poliklinik I, LMU Klinikum, LMU München, Munich, Germany; Medizinische Klinik und Poliklinik I, LMU Klinikum, LMU München, Munich, Germany; Medizinische Klinik und Poliklinik I, LMU Klinikum, LMU München, Munich, Germany; Medizinische Klinik und Poliklinik I, LMU Klinikum, LMU München, Munich, Germany; Medizinische Klinik und Poliklinik I, LMU Klinikum, LMU München, Munich, Germany; 1 Department of Cardiac, Thoracic Vascular Sciences and Public Health, University of Padua, Italy (G.M., F.A., S.C., A. Panza, G.T.).; 2 Department of Cardiology, Cardiology I, University Medical Center of the Johannes Gutenberg-University Mainz, Germany (K.-P.K., S.R., P. Lurz).; 3 Department of Cardiology, Heart Center, University of Cologne, Germany (J.S., C.I.).; 4 Clinic for General and Interventional Cardiology/Angiology, Herz- und Diabeteszentrum NRW, Universitätsklinik der Ruhr-Universität Bochum, Med. Fakultät OWL (Universität Bielefeld), Bad Oeynhausen, Germany (V.F., M. Kassar, V.R.).; 5 Department of Cardiology, University Heart and Vascular Centre Hamburg, Germany (B.K., D.K.).; 6 German Center of Cardiovascular Research (DZHK), Partner Site Hamburg/Kiel/Lübeck, Germany (B.K., D.K.).; 7 Department of Cardiology, University Heart Center Ulm, Germany (W.R., M. Kessler).; 8 Department of Cardiology, Inselspital, Bern University Hospital, University of Bern, Switzerland (M. Kassar, F.P.).; 9 Graduate School for Health Sciences, University of Bern, Switzerland (M. Kassar).; 10 Department of Cardiology, Heart Center, Zentralklinik Bad Berka, Germany (B.G., P. Lauten).; 11 Heart Valve Center, Cardio-Thoracic-Vascular Department, IRCCS San Raffaele Scientific Institute, Milan, Italy (P.D., F.M.).; 12 Cardiology Department, European Hospital Georges Pompidou, Université Paris Cité, France (P.A., N.K.).; 13 University Hospital Essen, University of Duisburg-Essen, West German Heart and Vascular Center, Department of Cardiology and Vascular Medicine, Germany (T.R., A.A.M.).; 14 Hospital Álvaro Cunqueiro, Vigo, Spain (M.B.-P., R.E.-L.).; 15 Department of Cardiology, Helios Klinikum Siegburg, Germany (P.B.).; 16 Department of Cardiology, Karolinska University Hospital, Stockholm, Sweden (A.R.).; 17 Medical Clinic III, University Hospital Tübingen, Germany (M.Z., T.G.).; 18 ASST Spedali Civili di Brescia and Department of Medical and Surgical Specialties, Radiological Sciences, and Public Health, University of Brescia, Italy (M.A., M.M.).; 19 Cardiology Department, Centre Hospitalier Universitaire De Lille, France (F. Vincent, E.V.B.).; 20 Division of Cardiology, Department of Internal Medicine III, University Hospital Heidelberg, Ruprecht-Karl University Heidelberg, Germany (P.S., M.H.K.).; 21 University Heart Center Freiburg/Bad Krozingen, Germany (M.G.W., C.B.).; 22 Heart Center Lucerne, Luzerner Kantonsspital, Switzerland (S.T., S.B.).; 23 Department of Cardiology, Guys and St Thomas NHS Trust, London, UK (J.G., T.P.).; 24 Department of Cardiology, Heart Center Leipzig at Leipzig University, Germany (H.T., T.K.).; 25 Cardiothoracic and Vascular Department, Azienda Ospedaliero-Universitaria Pisana, Pisa, Italy (A.S., M.D.C.).; 26 Department of Cardiology, Pulmonology and Angiology, Medical Faculty, Heinrich Heine University of Düsseldorf, Germany (F. Voss, A. Polzin).; 27 Department of Cardiology, IRCCS Policlinico San Donato, Milan, Italy (A.P.R., F.B.).; 28 Department of Cardiac Surgery, University Hospital Basel, University of Basel, Switzerland (T.S., T.N.).; 29 Department of Cardiology, University Hospital of León, Spain (T.B.-G., E.S.-M.).; 30 Medizinische Klinik und Poliklinik I, LMU Klinikum, LMU München, Germany (J.H., L.S.).; 31 German Center for Cardiovascular Research (DZHK), Partner Site Munich Heart Alliance, Germany (J.H., L.S.).

**Keywords:** cardiac catheterization, heart failure, hemodynamics, hypertension, pulmonary, tricuspid valve insufficiency

## Abstract

**BACKGROUND:**

Right heart catheterization plays a pivotal role in the preprocedural evaluation of patients considered for transcatheter tricuspid valve edge-to-edge repair. This study aimed to explore the potential impact of hemodynamic parameters obtained through right heart catheterization on patient-centered outcomes.

**METHODS:**

This study represents a subanalysis from the multicenter EuroTR registry (European Registry of Transcatheter Repair for Tricuspid Regurgitation). Patients with invasive hemodynamic data who underwent isolated transcatheter tricuspid valve edge-to-edge repair for significant tricuspid regurgitation were included. Outcomes of interest were a composite of 2-year all-cause death or hospitalization for heart failure (HFH) and a patient-centered composite of 6-month all-cause mortality, HFH, New York Heart Association class IV/worsening New York Heart Association class compared with baseline. Secondary outcome included postprocedural New York Heart Association class improvement.

**RESULTS:**

Seven hundred and eleven patients were included in the analysis. Two-year survival free from death and HFH was 63%. Optimal prognostic thresholds identified for death and HFH at 2 years were: mean pulmonary artery pressure≥32 mm Hg, pulmonary capillary wedge pressure (PCWP)≥20 mm Hg, and pulmonary vascular resistance≥5 wood units (WU). The early patient-centered composite outcome occurred in 25% of cases. PCWP≥20 mm Hg was independently associated with an early clinical deterioration (hazard ratio, 2.77 [95% CI, 1.47–5.28]; *P*<0.001) and with 2-year death/HFH (hazard ratio, 1.75 [95% CI, 1.03–3.02]; *P*=0.04). No invasive parameter was associated with residual tricuspid regurgitation ≥3+. New York Heart Association class improved significantly throughout the follow-up (*P*<0.001), although patients with elevated mean pulmonary artery pressure (*P*=0.04) or PCWP (*P*<0.01) experienced less symptomatic benefit.

**CONCLUSIONS:**

In patients undergoing transcatheter tricuspid valve edge-to-edge repair, invasive hemodynamics—especially elevated PCWP—are independently associated with early patient-centered outcomes and late adverse clinical events. Despite overall improvement of the functional status and no impact on residual tricuspid regurgitation, patients with higher mean pulmonary artery pressure or PCWP benefit less. These findings support the role of comprehensive right heart catheterization in preprocedural risk stratification.

**REGISTRATION:**

URL: https://clinicaltrials.gov; Unique identifier: NCT06307262.

**What is Known**

- Tricuspid regurgitation is increasingly prevalent, and transcatheter tricuspid valve edge-to-edge repair has emerged as the leading transcatheter therapy.
- Pulmonary hypertension is a key prognostic factor in transcatheter tricuspid valve edge-to-edge repair.

**What the Study Adds**

- Invasive hemodynamic measurements are linked to worse outcomes after transcatheter tricuspid valve edge-to-edge repair.
- Higher baseline mean pulmonary artery pressure or pulmonary capillary wedge pressure attenuates, but does not eliminate, postprocedural symptomatic improvement.
- Comprehensive right heart catheterization may enhance preprocedural risk stratification and guide postprocedural management.

**See Editorial by Huded and Chhatriwalla**

Tricuspid regurgitation (TR) is an increasingly prevalent condition in the aging population and is associated with poor functional status and adverse prognosis.^1,2^ In patients with severe TR, surgical intervention is often limited by elevated or prohibitive operative risk. Consequently, transcatheter treatment strategies have been introduced as less invasive therapeutic options.^3–5^ Among them, transcatheter tricuspid valve edge-to-edge repair (T-TEER) is the most frequently adopted approach, demonstrating feasibility, safety, and symptomatic improvement in carefully selected high-risk patients.^6,7^ However, overall mortality remains substantial, and both the optimal timing of intervention and patient selection criteria continue to generate debate. Notably, coexisting pulmonary hypertension has been linked to increased mortality in patients undergoing T-TEER.^8,9^ Given the challenges of accurately estimating pulmonary pressures using echocardiography in the presence of severe TR, right heart catheterization (RHC) plays a critical role in the preprocedural assessment.^4,5^ This study aims to assess the prognostic impact of invasively measured pulmonary hemodynamics and to determine whether specific threshold values can enhance risk stratification and support clinical decision-making in patients undergoing T-TEER.

## Methods

### Data Availability Statement

Data will not be available to others.

### Study Population

This study is a retrospective subanalysis derived from the EuroTR Registry (European Registry of Transcatheter Repair for Tricuspid Regurgitation), a multicenter, observational registry enrolling patients who underwent T-TEER for symptomatic TR between 2016 and 2024 across 26 European centers. Detailed methodology has been previously described.^9,10^ For this analysis, patients who underwent invasive hemodynamic assessment before the procedure were included. Moreover, to minimize potential confounding factors, patients undergoing concomitant mitral TEER were excluded. Procedural eligibility was determined by the local Heart Team at each participating site. Following informed consent, transcatheter tricuspid repair was performed using either the MitraClip/TriClip system (Abbott, Santa Clara, CA) or the PASCAL system (Edwards Lifesciences, Irvine, CA), in accordance with previously described techniques.^11,12^ This study adhered to the principles outlined in the Declaration of Helsinki and received proper ethical oversight (URL: https://clinicaltrials.gov/study/NCT06307262; Unique identifier: NCT06307262).

### Baseline Characteristics

Patient demographics and follow-up data were obtained from medical records or systematically collected at each participating center using standardized data collection forms. Transthoracic echocardiographic assessments were performed both before and after the procedure by experienced operators, in accordance with current guideline recommendations, as previously described.^13–15^ Both primary and secondary TR etiologies were included. Atrial secondary TR (A-STR) was defined as a right-atrium-to-right ventricle end-systolic area ratio of ≥1.5, provided that right ventricular function was preserved (tricuspid annular plane systolic excursion >17 mm).^10^ Preprocedural RHC was performed at the discretion of the treating center, aiming to capture the most representative hemodynamic status under optimal clinical conditions. Cardiac output (CO) was determined according to the internal standard of each center, and pulmonary vascular resistance (PVR) was calculated using the standard formula with mean pulmonary artery pressure (mPAP) and pulmonary capillary wedge pressure (PCWP): PVR=(mPAP−PCWP)/CO.^16^

### Study Outcomes

The outcomes of interest were a composite of death for all causes and heart failure hospitalization (HFH) at 2 years and a composite of early unfavorable patient-centered outcomes defined as death/HFH at 6 months or postprocedural New York Heart Association (NYHA) class worsening/persistent NYHA class IV.^4,17^ NYHA class change from baseline to follow-up was also evaluated.

### Statistical Analyses

Categorical variables are presented as absolute numbers with corresponding percentages, and group comparisons were performed using the χ^2^ test. Ordinal variables were assessed for trend across categories using the Mantel-Haenszel test for trend. The distribution of continuous variables was assessed using the Shapiro-Wilk test. Depending on data distribution, comparisons between groups were performed using either the Student *t* test or the Mann-Whitney *U* test. As the majority of continuous variables did not follow a normal distribution, all continuous data are reported as median with interquartile range (Q1–Q3) for consistency. Associations with patient-centered outcomes were explored using logistic regression models. Death and HFH at 2 years were analyzed using Cox proportional hazards regression (with associated statistical assumption assessed with Schoenfeld residuals) and illustrated using Kaplan-Meier survival curves. Discriminatory thresholds for continuous predictors were identified through receiver operating characteristic curve analysis and the Youden Index for patient-centered outcomes, and via maximally selected rank statistics for death-HFH at 2 years analyses. To assess potential nonlinear associations between continuous predictors and outcomes, logistic and Cox regression models incorporating restricted cubic splines with 3 knots were constructed. Considering the prognostic implication of each variable and its mathematical relationship, in cases of significant nonlinearity, covariate adjustments were applied for hemodynamic parameters, including PCWP, PVR, and CO. The statistical significance of spline components was evaluated using the Wald test. Variables demonstrating a significant association in the univariable analysis were subsequently included in multivariable models. To account for within-center correlation, each site was modeled as a cluster effect, and 95% CIs were calculated using robust Huber–White standard errors. To address the issue of multiple comparisons, a Bonferroni correction was applied, given the presence of 2 primary outcomes. To minimize multicollinearity, only 1 variable was selected among correlated parameters representing similar physiological domains. Interaction analysis was carried out to assess the influence on hazard estimate of the invasive parameters in different patients’ subgroups (age≥75 years, sex, A-STR, left ventricular ejection fraction≥35%, residual TR ≥3+). The study population was stratified according to the presence of zero, 1, 2, or 3 values exceeding the proposed thresholds for mPAP, PCWP, and PVR, and differences in survival were evaluated using the log-rank test. All statistical analyses were conducted using R software (version 4.4.3; R Foundation for Statistical Computing, Vienna, Austria) and Jamovi (The Jamovi Project, version 2.6.19.0). For all tests, a 2-tailed *P*<0.05 was considered significant.

## Results

Between 2016 and 2024, 711 patients (median age 81 [76, 84] years, median EuroSCORE 4.3 [2.4, 7.2]) underwent preprocedural RHC and comprised the analytic cohort for this study. Relevant baseline characteristics of the study population are shown in Table 1. Baseline differences for included and excluded patients are outlined in Table S1.

**Table 1.**
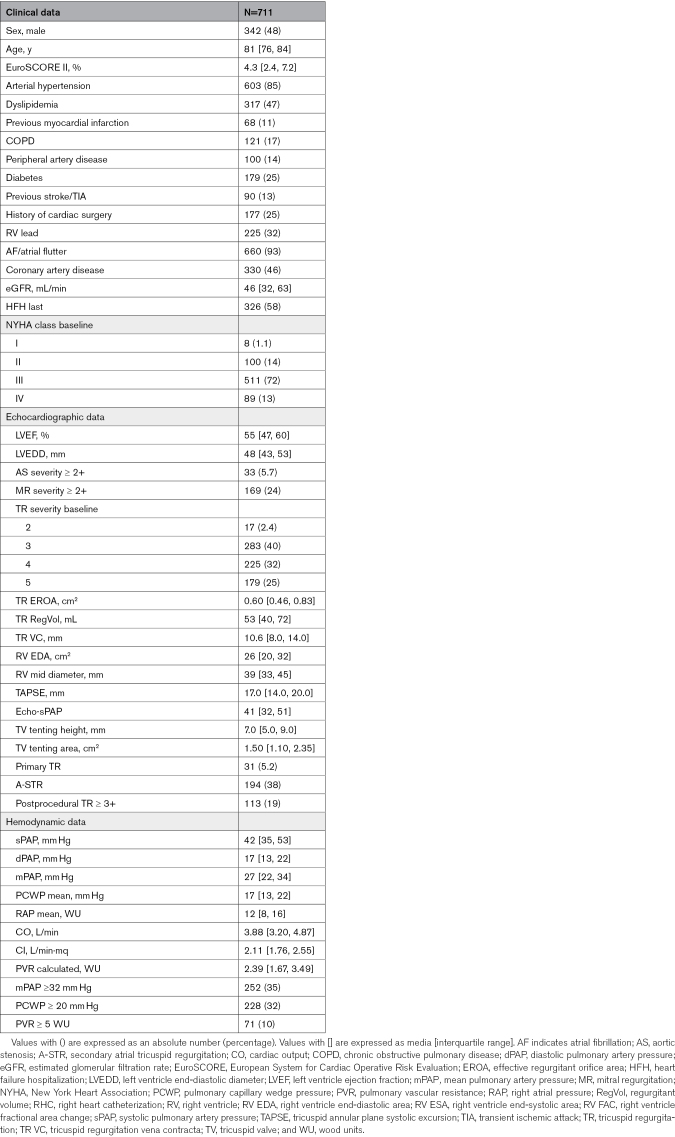
Baseline Characteristics of Study Population: Clinical, Echocardiographic, and Right Heart Catheterization

### Prognostic Impact of Invasive Hemodynamics in Patients Undergoing T-TEER

**Figure 1.**
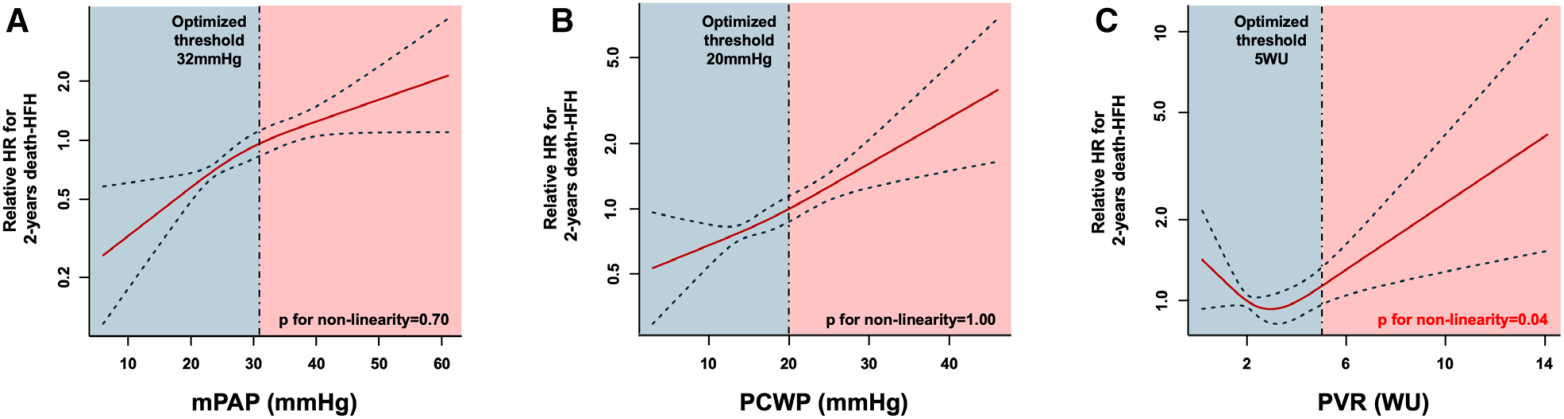
**Optimal thresholds and nature of relationship between mean pulmonary artery pressure (mPAP), pulmonary capillary wedge pressure (PCWP), pulmonary vascular resistance (PVR), and death-heart failure hospitalization (HFH) at 2 years. A**, Spline curve for mPAP. **B**, Spline curve for PCWP. **C**, Spline curve for PVR. Red line: relative hazard ratio (HR); blue dotted lines: CIs. CIs are wider in the extremes of PVR due to fewer observations. WU indicates wood units.

**Figure 2.**
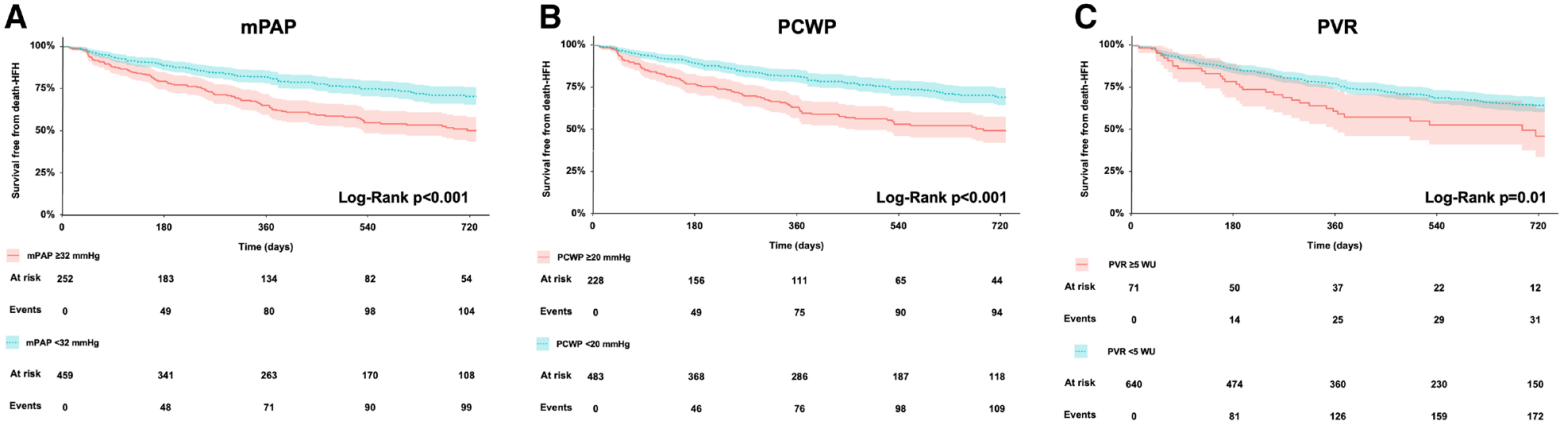
**Kaplan-Meier analysis survival free from death-heart failure hospitalization (HFH) at 2 years according to the extrapolated thresholds of mean pulmonary artery pressure (mPAP), pulmonary capillary wedge pressure (PCWP), and pulmonary vascular resistance (PVR). A**, Kaplan-Meier curve for mPAP. **B**, Kaplan-Meier curve for PCWP. **C**, Kaplan-Meier curve for PVR. WU indicates wood units.

**Figure 3.**
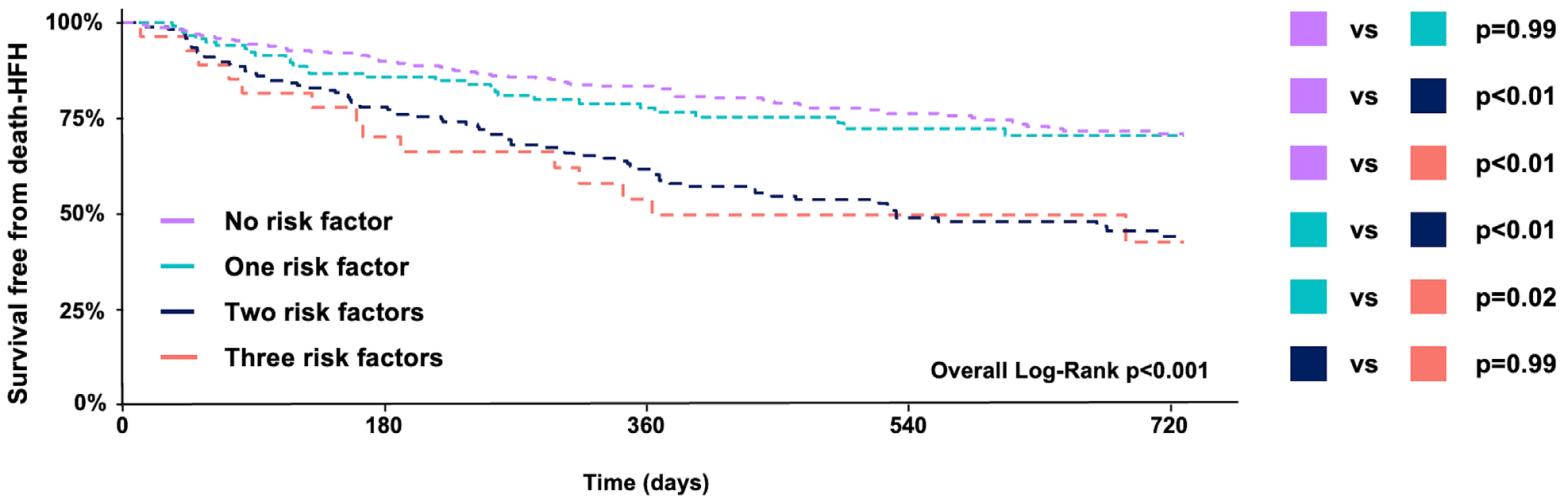
**Kaplan-Meier analysis survival free from death-heart failure hospitalization (HFH) at 2 years according to the presence of 0, 1, 2, or 3 parameters exceeding the optimized thresholds.** Thresholds are defined as follows: mean pulmonary artery pressure ≥32 mm Hg, (pulmonary capillary wedge pressure ≥20 mm Hg, pulmonary vascular resistance ≥5 wood units (WU). *P* values for pairwise comparison are shown at the right end of the figure.

Two-year survival free from death and HFH was 63%. All invasively derived hemodynamic parameters—except for PVR when modeled as a linear continuous variable—were significantly associated with the primary outcome (Table 2). PVR demonstrated a significant nonlinear relationship with adverse outcomes (*P*=0.04), characterized by a paradoxical increase in event rates at PVR values <2 wood units (WU), followed by a progressive rise in risk as PVR increased (Figure 1). The nonlinear component of the association was no longer significant when adjusted for PCWP and CO (Figure S1). No evidence of nonlinearity was observed for mPAP (*P*=0.70), PCWP (*P*=1.00), or CO (*P*=0.56). Threshold optimization using maximally selected rank statistics identified 32 mm Hg for mPAP, 20 mm Hg for PCWP, and 5 WU for PVR as the optimal cutoff values predictive of the 2-year death/HFH (Figure 1). All thresholds demonstrated significant prognostic discrimination in the study cohort (Figure 2; Table 2). Among all invasive parameters, PCWP ≥20 mm Hg, right atrial pressure, and CO remained independently associated with 2-year death or HFH in multivariable analysis (Table 3). The results of the stratification based on the cumulative presence of invasive parameters exceeding our threshold are shown in Figure 3. According to the log-rank analysis, the poorest prognosis was observed in patients with at least 2 parameters above the thresholds. Other noninvasive independent predictors of late outcomes were previous HFH, left ventricle end-diastolic diameter, tricuspid annular plane systolic excursion, and TR reduction. Notably, none of the invasive parameters proved to be an independent predictor of residual TR ≥3+ (Tables S2 and S3). Additionally, their prognostic impact was maintained regardless of TR reduction with no significant interaction detected (Table S4). No significant interactions were observed between invasive parameters and relevant subgroups, except for PCWP and A-STR (Table S4). Elevated PCWP has a more detrimental effect in patients with A-STR compared with patients with other etiologies (hazard ratio, 4.34 [95% CI, 2.06–9.13] for A-STR; hazard ratio, 1.75 [95% CI, 1.13–2.71] for non-A-STR; *P*_interaction_=0.04).

**Table 2.**
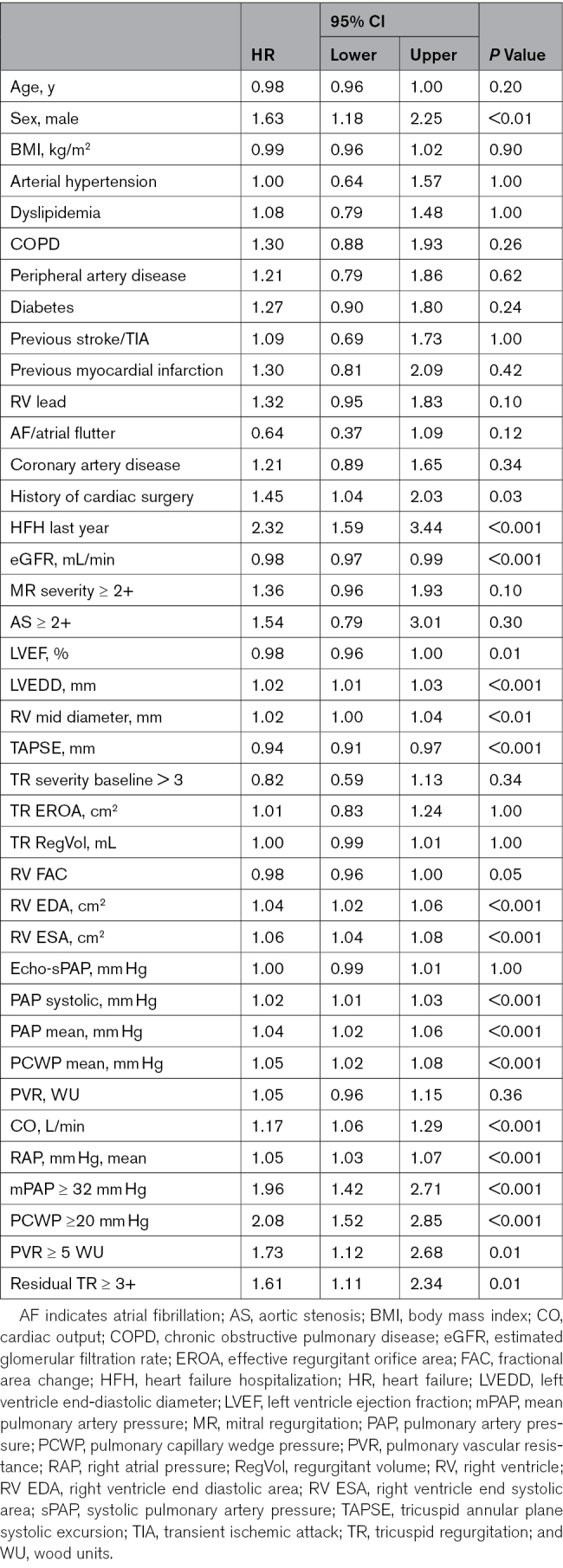
Univariate Analysis for Predictors of 2-Year All-Cause Death/HFH

**Table 3.**
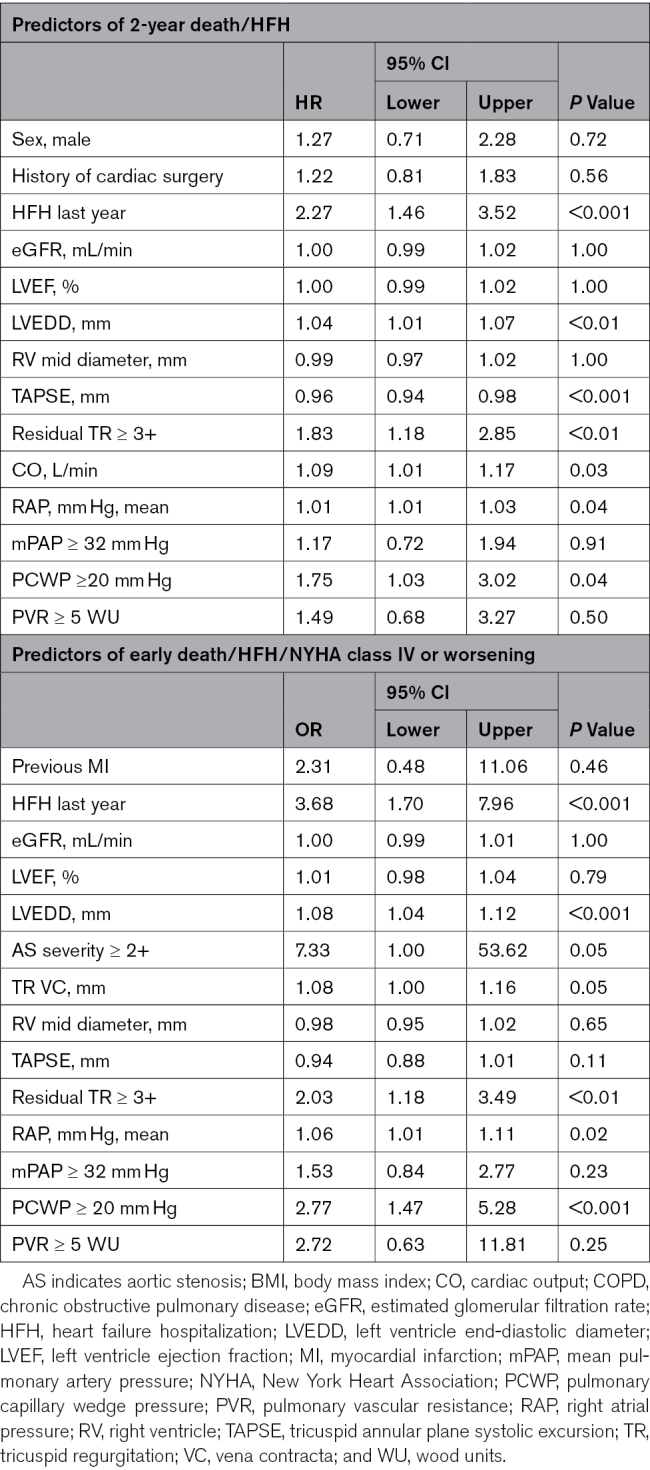
Multivariate Analysis for Predictors of 2-Year All-Cause Death/HFH and Early Death/HFH/NYHA IV or Worsening

### Early Unfavorable Patients-Centered Outcomes

Early unfavorable patient-centered outcomes occurred in 118 patients (24.6%). Receiver operating characteristic curve analysis identified optimal discriminatory thresholds of 32 mm Hg for mPAP and 20 mm Hg for PCWP in predicting early events (Figure S2). In contrast, receiver operating characteristic analysis for PVR did not reveal a meaningful threshold nor a significant association with early outcomes (Figure S2). PVR exhibited a significant nonlinear association with early unfavorable patient-centered outcomes (*P*=0.01), as illustrated in the spline-based analysis (Figure S3). To maintain consistency with the late clinical outcome analysis and based on visual interpretation of the spline curve, subsequent analyses were performed using a PVR threshold of 5 WU. A comprehensive list of univariable predictors for early patient-centered outcomes is presented in Table 4. Following multivariable adjustment, PCWP ≥20 mm Hg and right atrial pressure remained, among invasive parameters, independently associated with early unfavorable patient-centered outcomes (Table 3). Additional independent predictors included prior HFH, left ventricle end-diastolic diameter, and TR reduction (Table 3).

**Table 4.**
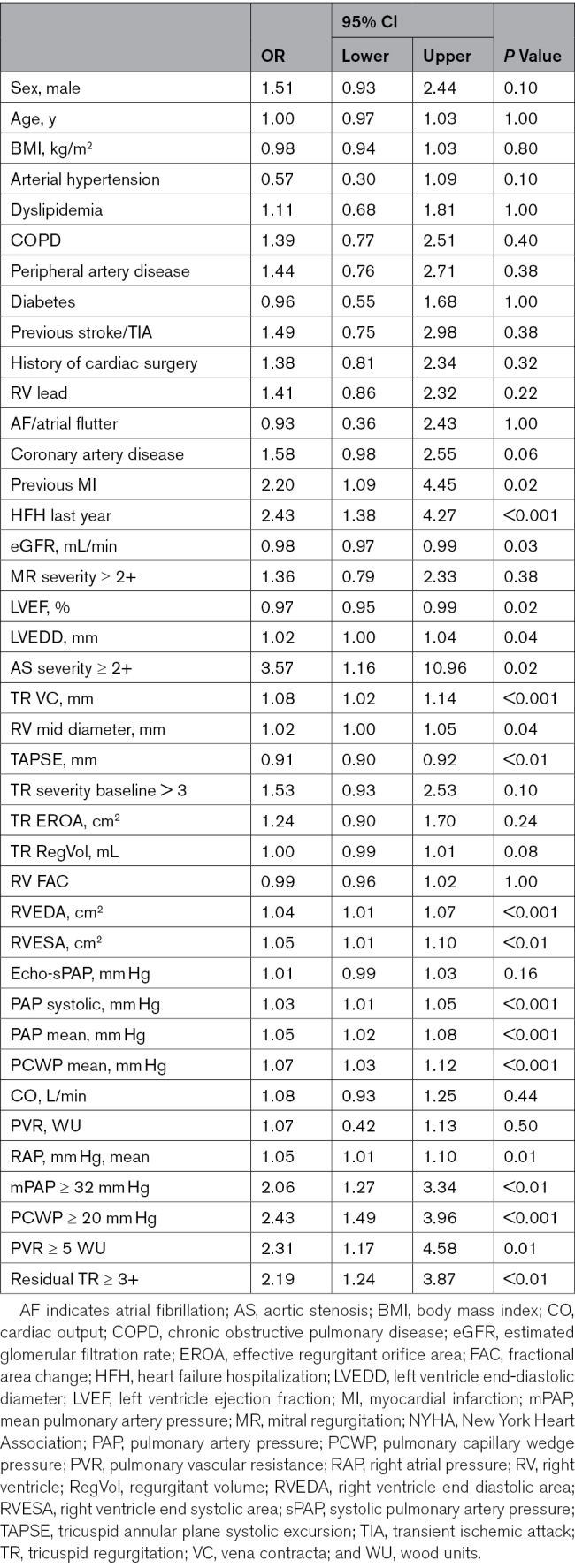
Univariate Analysis for Predictors of Composite Outcome of 6-Month All-Cause Mortality, HFH, NYHA Class IV Symptoms or Worsening NYHA Class

### Symptomatic Improvement Following T-TEER

**Figure 4.**
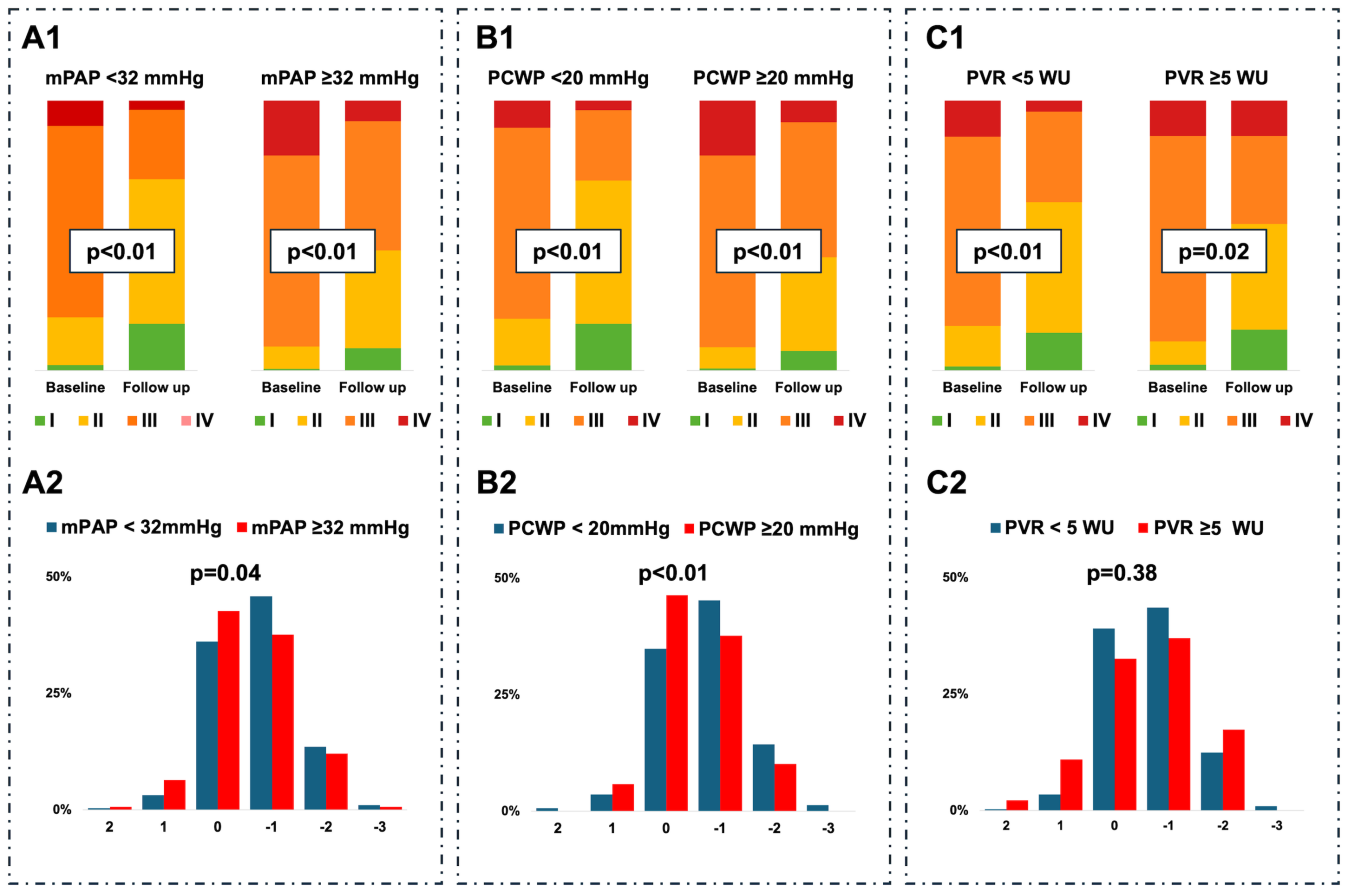
**Changes in New York Heart Association (NYHA) class after transcatheter tricuspid edge-to-edge repair (T-TEER). A1** and **A2**, Changes in NYHA class according to mean pulmonary artery pressure (mPAP). **B1** and **B2**, Changes in NYHA class according to pulmonary capillary wedge pressure (PCWP). **C1** and **C2**, Changes in NYHA class according to pulmonary vascular resistance (PVR). Paired sample analysis for NYHA class across the different subgroups from baseline to follow-up (panels above). Trend of NYHA change across the different subgroups from baseline to follow-up (panels below). Negative values represent improvement after T-TEER, positive values vice versa. Median follow-up: 271 (134, 392) days. WU indicates wood units.

T-TEER was associated with a reduction in NYHA functional class at follow-up (*P*<0.01; Figure S4). Patients with elevated mPAP or PCWP demonstrated a comparatively lower likelihood of NYHA class improvement than those with lower baseline values (Figure 4). Postprocedural NYHA improvement from baseline was consistently observed across all subgroups defined by invasive hemodynamic parameters (Figure 4).

## Discussion

In this multicenter retrospective study of patients undergoing T-TEER for clinically significant TR, we demonstrate that invasively derived hemodynamic parameters have strong prognostic value and can enhance risk stratification beyond current guideline-based diagnostic thresholds. The main findings are as follows: first, hemodynamic thresholds associated with adverse outcomes, particularly mPAP, PCWP, and PVR, were consistently higher than those proposed by the European Society of Cardiology (ESC) guidelines (mPAP>20 mm Hg, PCWP>15 mm Hg, PVR>2 WU) for the diagnosis of pulmonary hypertension, suggesting that T-TEER candidates may require tailored, procedure-specific risk definitions.^5,16^ Second, no single invasive parameter adequately reflects right heart pathophysiology on its own; comprehensive hemodynamic assessment is essential for accurate prognostication. Third, although patients with adverse hemodynamic profiles were more likely to experience early unfavorable events and showed attenuated symptomatic response, T-TEER still conferred meaningful clinical benefit, even in high-risk individuals. These findings are of particular relevance, as they address a patient population—those with relevant or precapillary pulmonary hypertension—that has been consistently excluded from major randomized trials.^18^ While ESC thresholds’ prognostic impact has proven valuable in left-sided heart disease, their relevance in TR is less certain.^8,19–21^ Both mPAP and PCWP demonstrated a linear association with risk, supporting the value of continuous scaling in outcome prediction. Our results reinforce the need to reconsider fixed diagnostic thresholds in favor of outcome-based, context-specific stratification. In contrast, the relationship between PVR and outcomes is more complex. While prior studies showed no association with PVR >2 WU, values exceeding 5 WU were prognostically relevant^8,9^. Our analysis supports these findings, as evidenced by the significant nonlinearity expressed by PVR. High CO and elevated PCWP, in addition to being mathematical determinants of low PVR, also are known as independent predictors of prognosis in patients undergoing T-TEER, as shown by our analysis and previous literature.^22,23^ This dual relationship acts as a confounder when evaluating the crude effect of PVR and represents the main determinant of its nonlinear association with outcomes. In addition to historical predictors (ie, HFH in the previous year), PCWP remained independently associated with both early and late outcomes, underscoring the influence of bi-ventricular heart failure in this population. This association is further supported by the independent predictive value of both left ventricle end-diastolic diameter and right atrial pressure for early and late outcomes. Whereas the left ventricle end-diastolic diameter reflects the contribution of LV dysfunction and dilation, right atrial pressure, although primarily elevated due to TR, also reflects pulmonary pressures as well as RV systolic and diastolic function. Taken together, these findings highlight how both left and RV dysfunction contribute to prognosis in this population.^24^ Notably, in patients with A-STR, the prognostic role of increased PCWP appears to be even more prominent. Those patients generally exhibit lower mPAP and PCWP and yet appear more vulnerable to the adverse prognostic impact of elevated PCWP compared with those non–A-STR.^10^ However, due to their interdependence, the isolated prognostic value of each invasive parameter is difficult to define and may be of limited clinical utility.^8^ When performed, RHC should be thorough. Risk stratification and treatment planning require an integrated assessment encompassing CO, PCWP, PVR, and mPAP, as more than 1 parameter exceeding the thresholds is needed to have the highest risk of death/HFH at follow-up. Whether these metrics serve only as prognostic markers or also represent modifiable therapeutic targets remains uncertain. Nonetheless, optimizing hemodynamics, particularly reducing PCWP, is a fundamental goal of TR management, and remains central to medical therapy.^25^ In patients with combined pre- and postcapillary pulmonary hypertension (PVR >5 WU), current ESC guidelines recommend a personalized approach.^16^ However, no therapies currently target the precapillary component in this population, and further investigation is warranted. Our findings provide a pathophysiological rationale to support such studies. In this context, future trials exploring targeted medical therapy or device-based unloading in selected patients, particularly those with elevated PCWP or borderline PVR, may further clarify the therapeutic potential of intervening on modifiable hemodynamic drivers. Finally, despite baseline hemodynamic severity, T-TEER led to consistent symptomatic improvement and TR reduction across all subgroups. In our cohort, none of the invasive parameters was independently associated with residual TR≥3+, whereas residual TR≥3+ itself emerged as an independent predictor of both early and late outcomes.^14^ The lack of interaction between TR≥3+ and invasive parameters suggests that the adverse prognostic impact of residual TR is consistently present, irrespective of baseline hemodynamic status. These findings emphasize the importance of achieving an optimal technical result, even in patients with unfavorable invasive parameters. Therefore, invasive hemodynamic measures should not be used to deny access to therapy, but rather to identify patients who may benefit from closer surveillance, intensified medical therapy, or early reevaluation following intervention.

### Limitations

This study has several limitations, primarily related to its retrospective and observational design. As such, it cannot provide definitive conclusions on the effect of T-TEER compared with medical therapy in these patients. The inclusion of only patients who underwent preprocedural RHC may have introduced selection bias, potentially enriching the study cohort with more symptomatic or clinically complex individuals. It is possible that patients undergoing RHC were more carefully evaluated or received optimized medical therapy, which could have played a role in outcomes independent of their invasive profile. As such, generalizability to the overall T-TEER population may be limited. Hemodynamic assessments were performed at the discretion of individual centers and were not centrally adjudicated, which may have introduced variability in measurement technique, timing, or interpretation. Similarly, echocardiographic evaluations, although conducted by experienced operators following current guideline recommendations, were not core lab–validated and may have been subject to interobserver variability. Finally, postprocedural hemodynamic data were not available in our cohort. Whether, and to what extent, T-TEER modifies each patient’s hemodynamic profile—and whether such changes carry prognostic significance—remains unknown. Further studies are warranted to clarify these aspects. Despite these limitations, the study benefits from a large, multicenter, contemporary cohort reflecting real-world clinical practice across 26 European tertiary centers. It includes standardized data collection, meaningful follow-up, and provides novel insights into both early and intermediate-term outcomes following T-TEER. These findings offer a valuable foundation to guide future prospective studies and inform refinement of clinical guidelines.

### Conclusions

In patients undergoing T-TEER, invasive hemodynamic assessment provides valuable prognostic information. PCWP were independently associated with both early unfavorable patient-centered outcomes and with death-HFH at 2 years. Notably, despite higher event rates, patients with adverse hemodynamic profiles still experienced meaningful improvements in functional status following T-TEER, underscoring the clinical value of the procedure even in high-risk individuals.

## Article Information

### Sources of Funding

Among the full cohort of patients in the registry, data collection for the Hamburg patients was supported by a grant from the German Heart Foundation.

### Disclosures

Dr Stolz received speaker honoraria from Edwards Lifesciences. Dr Kresoja reports travel expenses from Edwards Lifesciences. Dr von Stein received lecture honoraria from Edwards Lifesciences. Dr Rottbauer received speaker honoraria from Edwards Lifesciences and Abbott. Dr Denti served as Consultant for InnovHeart, Picardia, HVR Cardio, Approxima, and received speaker honoraria from Abbott and Edwards Lifesciences. Dr Rassaf received speaker honoraria and consulting fees from AstraZeneca, Bayer, Pfizer, and Daiichi Sankyo. Dr Barreiro-Perez received speaker fees from Abbott Vascular, Edwards Lifesciences, and Venus Medtech. Dr Adamo received consulting fees in the last 3 years from Abbott Structural Heart and Edwards Lifesciences. Dr Toggweiler has received personal honoraria from Medtronic, Boston Scientific, Biosensors, Abbott Vascular, Medira, Shockwave, Teleflex, atHeart Medical, Cardiac Dimensions, Polares Medical, Amarin, Sanofi, AstraZeneca, ReCor Medical, and Daiichi Sankyo, has received institutional research grants from Edwards Lifesciences, Boston Scientific, Fumedica, Novartis, Boehringer Ingelheim, and holds equity in Hi-D Imaging. Dr Marco Metra received consulting fees in the last 3 years from Abbott Structural Heart, AstraZeneca, Bayer, Boehringer Ingelheim, Edwards LifeSciences, and Roche Diagnostics. Dr Geisler received speaker honoraria/research grants from AstraZeneca, Bayer, Bristol Myers Squibb/Pfizer, Ferrer/Chiesi, Medtronic, and Edwards Lifesciences. None of them was related to this study. Dr Estévez-Loureiro received speaker fees from Abbott Vascular, Edwards Lifesciences, Boston Scientific, and Venus Medtech. Dr Maisano received a grant and research institutional support from Abbott, Medtronic, Edwards Lifesciences, Biotronik, Boston Scientific Corporation, NVT GmbH, Terumo, Venus, and consulting fees, honoraria, personal and institutional support from Abbott, Medtronic, Edwards Lifesciences, Xeltis, Cardiovalve, Occlufit, Simulands, Mtex, Venus, Squadra, Valgen, Royalty Income/IP Rights Edwards Lifesciences, and is a shareholder (including share options) of Magenta, Transseptal Solutions, 4Tech. Dr Fabien Praz received travel expenses from Edwards Lifesciences, Abbott Vascular, Polares Medical, Medira, and Siemens Healthineers. Dr Kessler received speaker honoraria from Edwards Lifesciences and Abbott. Dr Kalbacher has received personal fees from Abbott Medical, Edwards Lifesciences, Pi-Cardia Ltd, and Medtronic Inc. Dr Rudolph received research grants from Abbott Medical, Boston Scientific, and Edwards Lifesciences. Dr Iliadis received consultant fees and travel expenses from Abbott Medical and Edwards LifeSciences. Dr Sticchi served on the advisory board for Edwards Lifesciences. Dr Lurz received institutional grants from Edwards Lifesciences and honoraria from Innoventrics. Dr Hausleiter reports research grant support and speaker honoraria from Edwards Lifesciences. Dr Tarantini received a speaker fee for Abbott Vascular and Edwards Lifesciences. Dr Mahabadi received speaker fees and advisory boards: Amgen, Berlin Chemie, Daiichi Sankyo, Edwards Lifesciences, Novartis, and Sanofi. Research funding: Daiichi Sankyo, Edwards Lifesciences, all outside the submitted work. Dr Nestelberger has received research support from the Swiss National Science Foundation (P400PM_191037/1), the Prof. Dr. Max Cloëtta Foundation, the Margarete und Walter Lichtenstein-Stiftung (3MS1038), the Freiwillige Akademische Gesellschaft Basel, the Stiftung kardiovaskuläre Forschung Basel, the University of Basel, the University Hospital Basel, as well as speaker/consulting honoraria or research support from Edwards Lifesciences, Pronova Medical, Meril, Boston Scientific, Medtronic, Abbott, Beckman Coulter, Bayer, Ortho Clinical Diagnostics, and Orion Pharma, outside the submitted work. The other authors report no conflicts.

### Supplemental Material

Tables S1–S4

Figures S1–S4

## Appendix

EuroTR Investigators: Karl-Philip Rommel, MD (Department of Cardiology, Cardiology I, University Medical Center of the Johannes Gutenberg-University Mainz, Mainz, Germany); Ralph Stephan von Bardeleben, MD (Department of Cardiology, Cardiology I, University Medical Center of the Johannes Gutenberg-University Mainz, Mainz, Germany); Roman Pfister, MD (Department of Cardiology, Heart Center, University of Cologne, Cologne, Germany); Stephan Baldus, MD (Department of Cardiology, Heart Center, University of Cologne, Cologne, Germany); Philipp von Stein, MD (Department of Cardiology, Heart Center, University of Cologne, Cologne, Germany); Muhammed Gerçek, MD (Clinic for General and Interventional Cardiology/Angiology, Herz- und Diabeteszentrum NRW, Universitätsklinik der Ruhr-Universität Bochum, Med. Fakultät OWL (Universität Bielefeld), Bad Oeynhausen, Germany); Felix Rudolph, MD (Clinic for General and Interventional Cardiology/Angiology, Herz- und Diabeteszentrum NRW, Universitätsklinik der Ruhr-Universität Bochum, Med. Fakultät OWL (Universität Bielefeld), Bad Oeynhausen, Germany); Hazem Omran, MD (Clinic for General and Interventional Cardiology/Angiology, Herz- und Diabeteszentrum NRW, Universitätsklinik der Ruhr-Universität Bochum, Med. Fakultät OWL (Universität Bielefeld), Bad Oeynhausen, Germany); Sebastian Ludwig, MD (Department of Cardiology, University Heart and Vascular Centre Hamburg, Hamburg, Germany; German Center of Cardiovascular Research (DZHK), Partner Site Hamburg/Kiel/Lübeck, Germany); Christoph Pauschinger, MD (Department of Cardiology, University Heart and Vascular Centre Hamburg, Hamburg, Germany; German Center of Cardiovascular Research (DZHK), Partner Site Hamburg/Kiel/Lübeck, Germany); Leonhard-Moritz Schneider, MD (Department of Cardiology, University Heart Center Ulm, Ulm, Germany); Matthias Gröger, MD (Department of Cardiology, University Heart Center Ulm, Ulm, Germany); Dominik Felbel, MD (Department of Cardiology, University Heart Center Ulm, Ulm, Germany); Carsten Salomon, MD (Department of Cardiology, Heart Center, Zentralklinik Bad Berka, Bad Berka, Germany); Harald Lapp, MD (Department of Cardiology, Heart Center, Zentralklinik Bad Berka, Bad Berka, Germany); Quentin de Baynast, MD (Cardiology Department, Centre Hospitalier Universitaire De Lille, Lille, France); Alain Berrebi, MD (Cardiology Department, Centre Hospitalier Universitaire De Lille, Lille, France); Florian Schindhelm, MD (University Hospital Essen, University of Duisburg-Essen, West German Heart and Vascular Center, Department of Cardiology and Vascular Medicine, Essen, Germany); Berenice Caneiro-Queija, MD (Hospital Álvaro Cunqueiro, Vigo, Spain); Julio Echarte-Morales, MD (Hospital Álvaro Cunqueiro, Vigo, Spain); Andreas Goldschmied, MD (Medical Clinic III, University Hospital Tübingen, Tübingen, Germany); Edoardo Pancaldi, MD (ASST Spedali Civili di Brescia and Department of Medical and Surgical Specialties, Radiological Sciences, and Public Health, University of Brescia, Brescia, Italy); Elisa Pezzola, MD (ASST Spedali Civili di Brescia and Department of Medical and Surgical Specialties, Radiological Sciences, and Public Health, University of Brescia, Brescia, Italy); Mauro Massussi, MD (ASST Spedali Civili di Brescia and Department of Medical and Surgical Specialties, Radiological Sciences, and Public Health, University of Brescia, Brescia, Italy); Laura Lupi, MD (ASST Spedali Civili di Brescia and Department of Medical and Surgical Specialties, Radiological Sciences, and Public Health, University of Brescia, Brescia, Italy); Natacha Rousse, MD (Cardiology Department, Centre Hospitalier Universitaire De Lille, Lille, France); Samy Aghezzaf, MD (Cardiology Department, Centre Hospitalier Universitaire De Lille, Lille, France); Sirin Bakhtari, MD (Cardiology Department, Centre Hospitalier Universitaire De Lille, Lille, France); Norbert Frey, MD (Department of Internal Medicine III, Division of Cardiology, University Hospital Heidelberg, Ruprecht-Karl University Heidelberg, Heidelberg, Germany); Martin Kraus, MD (Department of Internal Medicine III, Division of Cardiology, University Hospital Heidelberg, Ruprecht-Karl University Heidelberg, Heidelberg, Germany); Dirk Westermann, MD (University Heart Center Freiburg/Bad Krozingen, Bad Krozingen, Germany); Matteo Mazzola, MD (Cardiothoracic and Vascular Department, Azienda Ospedaliero-Universitaria Pisana, Pisa, Italy); Cristina Giannini, MD (Cardiothoracic and Vascular Department, Azienda Ospedaliero-Universitaria Pisana, Pisa, Italy); Anke von Peter, MD (Department of Cardiac Surgery, University Hospital Basel, University of Basel, Basel, Switzerland); Julia Novotny, MD (Department of cardiac, thoracic vascular sciences and public health, University of Padua, Padua, Italy); Ludwig T. Weckbach, MD (Medizinische Klinik und Poliklinik I, LMU Klinikum, LMU München, Munich, Germany); Thomas J. Stocker, MD (Medizinische Klinik und Poliklinik I, LMU Klinikum, LMU München, Munich, Germany); Kaspar Volz, Cand. Med (Medizinische Klinik und Poliklinik I, LMU Klinikum, LMU München, Munich, Germany); Katalin Berschiminski, Cand. Med. (Medizinische Klinik und Poliklinik I, LMU Klinikum, LMU München, Munich, Germany); Philipp Doldi, MD (Medizinische Klinik und Poliklinik I, LMU Klinikum, LMU München, Munich, Germany); Hannah Kempton, MD (Medizinische Klinik und Poliklinik I, LMU Klinikum, LMU München, Munich, Germany); Dario Grassini, MD (Medizinische Klinik und Poliklinik I, LMU Klinikum, LMU München, Munich, Germany).

## Supplementary Material
