# Supplementary material for: Invasive Hemodynamics and Risk Stratification in T-TEER: Moving Beyond ESC Thresholds - EuroTR Registry Insights
Source: Circ Cardiovasc Interv. 2025 Nov 14;19(1):e015964. doi: 10.1161/CIRCINTERVENTIONS.125.015964 (PMC12825785; doi:10.1161/CIRCINTERVENTIONS.125.015964)
Supplement: Supplementary file 1 [file hcv-19-e015964-s001.pdf]

SUPPLEMENTAL MATERIAL

Supplemental Figures

Figure S1: Spline curve analysis for death-HFH at 2 years for PVR adjusted for PCWP and CO. *CO: cardiac output; PCWP: pulmonary capillary wedge pressure, PVR: pulmonary vascular resistance; HFH: heart failure hospitalization. .*

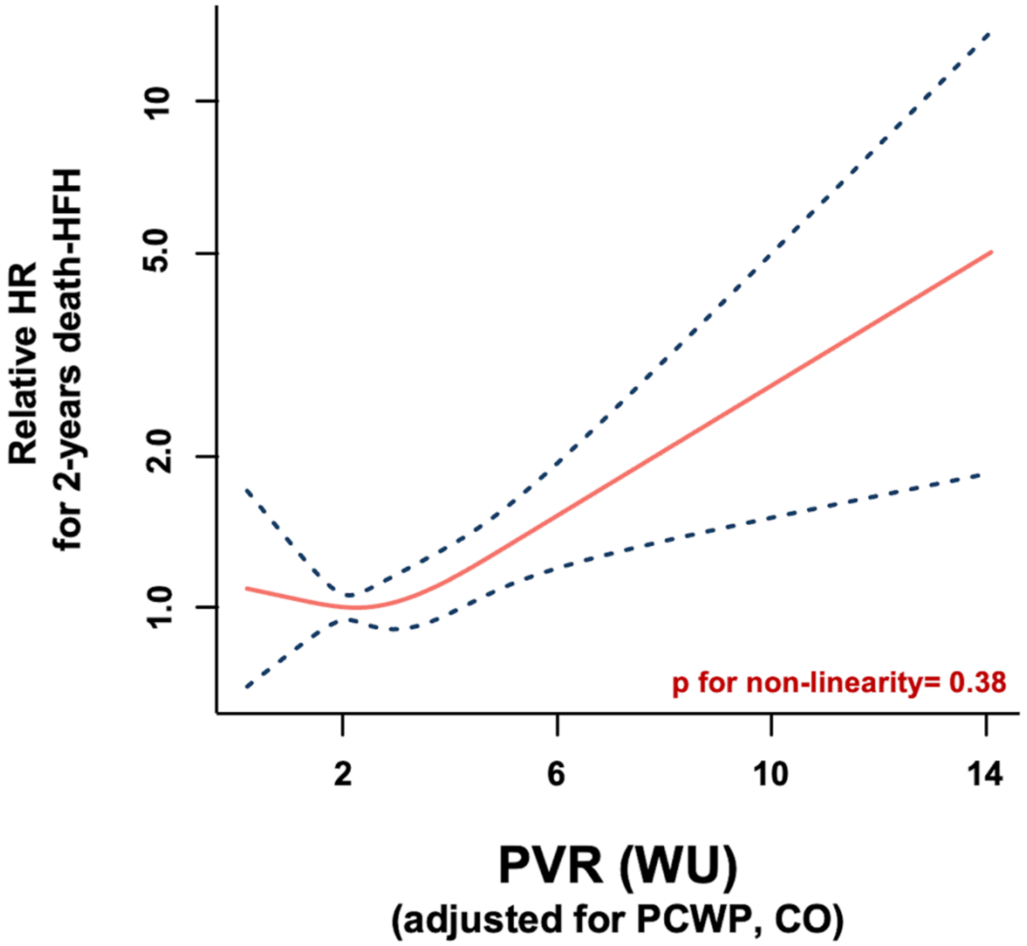

Figure S2: ROC curve analysis for early unfavorable patients-centered outcomes. *mPAP: mean pulmonary artery pressure; PCWP: pulmonary capillary wedge pressure; PVR: pulmonary vascular resistance; AUC: area under the curve.*

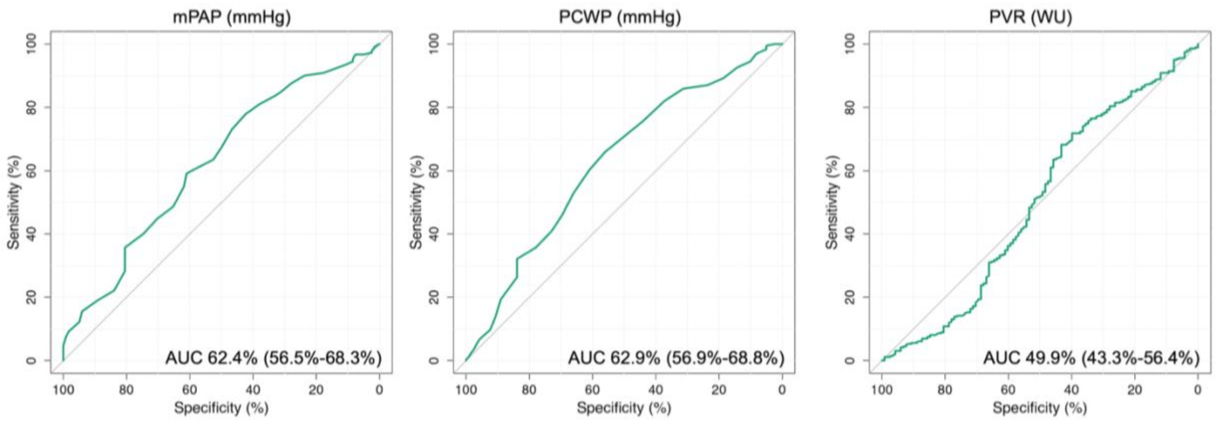

Figure S3: Non-linear relationship between PVR and early unfavorable patients-centered outcomes. Unadjusted spline curve according to PVR values. Reference HR is set at PVR= 2 WU. Red line: relative HR; blue dotted lines: confidence intervals. Confidence intervals are wider in the extremes of PVR due to fewer observation. *PVR: pulmonary vascular resistance.*

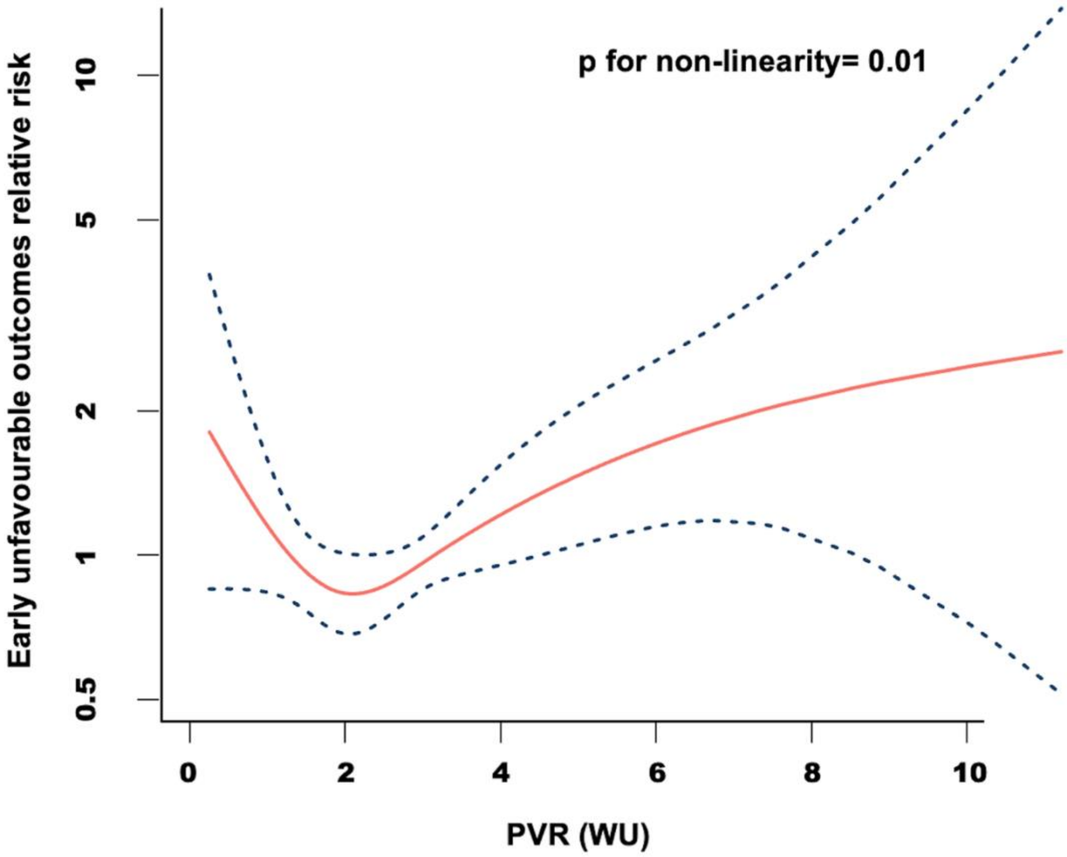

Figure S4: Changes in NYHA class after T-TEER in the overall study population. Median follow up of 361 (118, 666) days. *NYHA: New York Heart Association; T-TEER: transcatheter tricuspid edge-to-edge repair.*

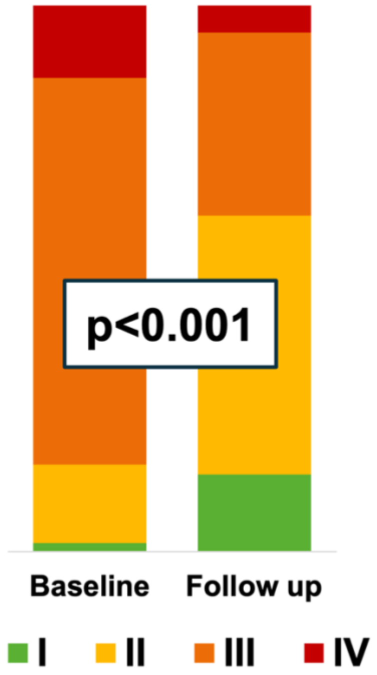

Supplemental Tables

***Table S1*: Baseline characteristics between study population (i.e. patients that underwent invasive hemodynamic assessment prior to the procedure) and excluded patients.**

Clinical data	*Overall (n= 2798)*	*RHC - (n= 2087)*	*RHC + (n= 711)*	*p*
Male sex (n= 2786)	1260	918 (44)	342 (48)	0.07
Age, y (n= 2789)	80.0 [76.0, 83.0]	80 [75, 83]	81 [76, 84]	0.05
EuroSCORE II, % (n= 2317)	4.5 [2.6-, .6]	4.6 [2.6, 7.8]	4.3 [2.4, 7.2]	0.08
Arterial hypertension (n= 2610)	2060 (78.9)	1457 (77)	603 (85)	<.01
Dyslipidemia (n= 2576)	1266 (49.1)	949 (50)	317 (47)	0.16
Previous myocardial infarction (n= 2486)	252 (10.1)	184 (9.8)	68 (11)	0.31
COPD (n= 2665)	422 (15.8)	301 (15)	121 (17)	0.31
Peripheral artery disease (n= 1665)	205 (12.3)	105 (11)	100 (14)	0.04
Diabetes mellitus (n= 2590)	606 (23.4)	427 (23)	179 (25)	0.16
Previous stroke/TIA (n= 2000)	240 (12.0)	150 (12)	90 (13)	0.47
History of cardiac surgery (n= 2679)	750 (28.0)	573 (29)	177 (25)	0.03
RV lead (n= 2794)	778 (27.8)	533 (27)	225 (32)	0.01
AF/atrial flutter (n= 2750)	2483 (90.3)	1823 (89)	660 (93)	0.01
Coronary artery disease (n= 2734)	1118 (40.9)	788 (39)	330 (46)	<.01
eGFR, mL/min (n= 2411)	44.4 [32.0, 59.0]	44 [32, 58]	46 [32, 63]	0.13
HFH last (n= 1347)	735 (54.6)	409 (52)	326 (58)	0.02
NYHA class baseline (n= 2774)				0.31
I	39 (1.4)	31 (1.5)	8 (1.1)	
II	446 (16.1)	346 (17)	100 (14)	
III	1964 (70.8)	1453 (70)	511 (72)	
IV	235 (11.7)	236 (11)	89 (13)	
Echocardiographic data				
LVEF, % (n= 2638)	55.0 [47.0, 60.0]	55 [47, 60]	55 [47, 60]	0.15
LVEDD, mm (n= 2421)	47.5 [43.0-, 3.0]	48 [43, 53]	48 [43, 53]	0.97
AS severity ≥ 2+ (n= 1659)	89 (5.4)	56 (5.2)	33 (5.7)	0.66
MR severity ≥ 2+ (n= 2621)	703 (26.8)	534 (28)	169 (24)	0.08
TR severity baseline (n= 2716)				<.01
2	69 (2.5)	52 (2.6)	17 (2.4)	
3	1198 (44.1)	915 (45)	283 (40)	
4	893 (32.9)	668 (33)	225 (32)	
5	556 (20.5)	377 (19)	179 (25)	
TR EROA, cm^2 (n= 2253)	0.6 [0.4, 0.8]	0.55 [0.40, 0.78]	0.60 [0.46, 0.83]	<0.01
TR RegVol, mL (n= 1936)	48.0 [36.0, 65.0]	46 [35, 62]	53 [40, 72]	<0.01

TR VC, mm (n= 2377)	10.0 [8.0, 14.0]	10.0 [8.0, 14.0]	10.6 [8.0, 14.0]	0.04
RV EDA, cm^2 (n= 1461)	25.6 [10.0, 31.6]	25 [20, 32]	26 [20, 32]	0.74
RV mid diameter, mm (n= 1848)	39.0 [33.0, 45.0]	39 [34, 46]	39 [33, 45]	0.20
TAPSE, mm (n= 2458)	17.0 [14.0, 20.0]	17.0 [14.0, 20.0]	17.0 [14.0, 20.0]	0.67
Echo-sPAP (n= 2462)	42.0 [33.0, 51.9]	42 [34, 52]	41 [32, 51]	0.08
TV tenting height, mm (n= 1222)	7.2 [5.0, 10.0]	8.0 [5.8, 10.0]	7.0 [5.0, 9.0]	<.01
TV tenting area, cm^2 (n= 1139)	1.6 [1.1, 2.4]	1.70 [1.10, 2.40]	1.50 [1.10, 2.35]	0.37
Post procedural TR ≥ 3+ (n= 2545)	440 (17.3)	327 (17)	113 (19)	0.24

Values with ( ) are expressed as absolute number (percentage). Values with [ ] are expressed as media [interquartile range]. RHC: right heart catheterization; COPD: chronic obstructive pulmonary disease; RV: right ventricle; AF: atrial fibrillation; eGFR: estimated glomerular filtration rate; HFH: heart failure hospitalization; NYHA: New York Heart Association; LVEF: left ventricle ejection fraction; LVEDD: left ventricle end-diastolic diameter; AS: aortic stenosis; MR: mitral regurgitation; TR: tricuspid regurgitation; EROA: effective regurgitant orifice area; TR VC: tricuspid regurgitation vena contracta; RV FAC: right ventricle fractional area change; RV EDA: Right ventricle end diastolic area; RV ESA: Right ventricle end systolic area; TAPSE: tricuspid annular plane systolic excursion; sPAP: systolic pulmonary artery pressure; TV: tricuspid valve; WU: wood unit.

Table S2: Univariable analysis for residual TR≥3+.

	OR	95% CI		p
mPAP (each mmHg)	0.98	0.96	1.00	0.11
mPAP ≥32 mmHg:	0.71	0.45	1.01	0.12
PCWP (each mmHg)	1.02	0.98	1.05	0.34
PCWP ≥ 20 mmHg	1.23	0.80	1.88	0.35
**PVR (each WU)**	**0.79**	**0.68**	**0.92**	**<0.01**
PVR ≥ 5 WU	0.62	0.29	1.35	0.23
CO (l/min)	0.95	0.83	1.10	0.51

mPAP: mean pulmonary artery pressure; PCWP: pulmonary capillary wedge pressure; PVR: pulmonary vascular resistance; CO: cardiac output; TR: tricuspid regurgitation.

Table S3: Multivariable adjustment for residual TR≥3+. Adjustment for PVR (each WU).

**For PVR (each mmHg)**				
	**OR**	**95% CI**		**P**
Sex (female = 0, male = 1)	0.86	0.39	1.89	0.71
Age (years)	0.98	0.93	1.05	0.69
History of Cardiac surgery	1.66	0.75	3.66	0.21
LVEF (%)	0.99	0.97	1.03	0.85
Baseline TR>3	1.67	0.69	4.06	0.26
Baseline AS≥2+	5.07	0.90	28.7	0.07
Vena contracta (mm)	1.09	1.00	1.18	0.05
Rvmid diameter (mm)	0.98	0.94	1.02	0.29
RV lead	1.24	0.57	2.71	0.58
Coaptation Gap (mm)	1.20	1.04	1.38	0.01
TV Tenting area (cm2)	1.94	1.43	2.63	<.001
PVR (each mmHg)	0.89	0.69	1.14	0.35

PVR: pulmonary vascular resistance; LVEF: left ventricle ejection fraction; TR: tricuspid regurgitation; AS: aortic stenosis; RV: right ventricle, TV: tricuspid valve

Table S4: Interaction analysis for death-HFH at 2 years according to different patient's subgroups. Values shown refers to p for interaction.

	**Age ≥ 75 y**	**Sex**	**A-STR**	**LVEF ≥35%**	**Residual TR ≥3+**
mPAP ≥ 32 mmHg	0.20	1.00	0.46	1.00	1.00
PCWP ≥ 20 mmHg	0.70	1.00	**0.04**	0.30	0.60
PVR ≥ 5 WU	1.00	0.34	0.30	0.48	0.26

A-STR: secondary atrial tricuspid regurgitation, LVEF: left ventricle ejection fraction TR: tricuspid regurgitation, mPAP: mean pulmonary artery pressure; PCWP: pulmonary capillary wedge pressure; PVR: pulmonary vascular resistance; ESC: European Society of Cardiology
